# Alternative use of phage display: phage M13 can remain viable in the intestines of poultry without causing damage

**DOI:** 10.1186/s13568-022-01407-9

**Published:** 2022-06-02

**Authors:** Fabiana de Almeida Araújo Santos, Edson Campos Valadares Junior, Luiz Ricardo Goulart, Pedro Lucas Figueiredo Nunes, Eliane Pereira Mendonça, Lúcio Vilela Carneiro Girão, Aline Santana da Hora, Thatiana Bragine Ferreira, Luciana Machado Bastos, Alessandra Aparecida Medeiros-Ronchi, Belchiolina Beatriz Fonseca

**Affiliations:** 1 Faculdade de Medicina Veterinária Universidade Federal de Uberlândia, Uberlândia, Brazil; 2 Instituto de Biotecnologia da Universidade Federal de Uberlândia, Uberlândia, Brazil; 3 Pós-Graduação em Medicina Tropical e Infectologia da Universidade Federal do Triângulo Mineiro, Uberaba, Brazil

**Keywords:** Control, ELISA, Peptides, Chickens, Chicken gastrointestinal tract

## Abstract

Phage display (PD) is a tool for developing new molecules to control pathogens. Peptides selected by PD are commonly synthesised and tested, but the use of phage M13 displaying the selected peptides as a direct biding in the intestinal tract has not yet been tested. This study evaluated whether phage M13 can remain viable in the chicken gastrointestinal tract and whether it causes injury or humoral immune response. We inoculated phage M13 or *E. coli* ER2738 (ECR) infected with M13 into birds at different ages. We found the virus in faeces at 5 or 13 days after inoculation, just when it infected the ECR. The presence of phage M13 or ECR did not result in gut injuries and had no impacts on weight gain and bird health. Furthermore, the levels of IgY were similar in all treatments, which indicates that the virus can be used in chicken until 42 days without being recognised by the immune system. This work provides a scientific basis for the use of PD as a tool in numerous applications to control different pathogens.

**Supplementary Information:**

The online version contains supplementary material available at 10.1186/s13568-022-01407-9.

## Key Points

- Phage M13 remains viable in the bird's intestine if inoculated with *E. coli* ER2738.
- Bacteriophage M13 does not damage the chicken gut.
- Phage M13 remains in the gut without leading to a humoral response up to 42 days.

**Supplementary Information:**

The online version contains supplementary material available at 10.1186/s13568-022-01407-9.

## Introduction

The search for antibiotics replacement is still an important topic. The increase in the use of antibiotics in poultry production is correlated to an increased antibiotic resistance of bacteria (Muaz et al. 2018), leading to complications in the treatment of bacterial diseases in humans and animals. In this sense, several alternatives to antibiotics are used in poultry production with varying success, such as probiotics, prebiotics, symbiotics, essential oils, phytotherapy, organic acids, enzymes, bacteriophages and peptides (Gadde et al. 2017). In addition, the use and study of new vaccines are critical tools to reduce the among of antibiotics in poultry production.

The phage display (PD) technique can contribute to the replacement of antibiotics; it is used to select, characterise and identify peptides (Saw and Song 2019). The bacteriophage M13 is a filamentous phage that displays peptides linked to the minor coat protein III gene. Based on the vast library displayed in M13, peptides can be selected and purified; a high amount of the bacteriophage has been replicated in a modified *E. coli* (Barbas et al. 2001). This provides high-affinity peptides, which can be employed in the development of peptides to vaccines (Ma et al. 2019) and in therapeutics strategies (Mimmi et al. 2019).

After the selection by PD, the peptides displayed by phage M13, are sequenced, characterised and synthesised for later use. In the poultry industry, oral vaccination or treatment can be adequate because of the group approach. Additionally, some microorganisms, such as intestinal *Salmonella, Campylobacter* and *Eimeria*, are usually limited to the gastrointestinal tract in birds, which requires prevention or oral therapy. In the context of vaccines, in some cases, peptides cannot stimulate the immune response because of the limitations concerning immunogenicity (Malonis et al. 2020). Therefore, although treatment with antimicrobial peptides can be efficient (Wang et al. 2016), alternative methods should be developed.

According to a previous study, the epitopes expressed in bacteriophages selected by PD can stimulate the immune response (Díaz-Valdés et al. 2011). However, the use of the bacteriophage M13 displaying selected peptides has not yet been investigated in the poultry gut, and therefore, we do not know whether bacteriophage M13 can survive in the gastrointestinal tract and be innocuous to poultry. A deeper knowledge of this issue can facilitate other studies on phage M13 displaying the peptides. Being a larger structure than the purified peptide, phage M13 displaying selected peptides may stimulate the local immune response and/or bind to microorganisms more successfully. In this context, we evaluated the viability and innocuity of the wild M13 bacteriophage in the intestine of chickens, with the aim to obtain an alternative for future studies in which these phages are used in therapies or vaccines.

## Methods

A total of 140 chicks (Hy-Line w-36®) from an industrial incubator were grown in the Ornitopathology Laboratory of the Federal University of Uberlândia, Brazil. The animals were housed on the same day of birth and kept until 42 days of age on the floor in previously autoclaved wood shavings. Water and food were provided at libitum during the entire rearing period; temperature and humidity were controlled according to the age. The final density was 7 birds/m^2^. The nutritional level of the feed is shown in Additional file 1: Table S1 and S2.

Management, sampling and euthanasia were carried out following the guidelines of the Committee on Ethics in the Use of Animals of the Federal University of Uberlândia (nº 118/18).

### Inoculum preparation and titration

The inoculum was prepared after the incubation of one colony of *Escherichia coli* ER2738 (ECR) (New England Biolabs) at 37 °C in *Luria Bertani*—(LB—Tryptone 10 g/L, yeast extract 5 g/L, NaCl 10 g/L) (Kasvi) with tetracycline (Sigma Chemical Co., 20 mg/mL) under agitation until the *earlylog* (OD600 ~ 0.3). Part of the inoculum was stored, and the other part was inoculated with the 10 log PFU of phage M13 (New England Biolabs) incubated at 37 °C for 4 h under agitation. The culture was centrifuged at 15,000 × *g* for 10 min, and the pellet containing ECR infected by the M13 phage was used as inoculum. The supernatant was transferred to a tube containing PEG/NaCl (20% polyethylene glycol 8000, Fluka, and 2.5 M NaCl Neon—sterile solution), incubated at 4 °C for 16 h and centrifuged. Finally, the phage pellet was resuspended with sterile PBS and used as phage inoculum.

Infected and non-infected bacteria were quantified on an LB agar plate after several dilutions. For titrating of the phage M13, the ECR was previously prepared in LB and incubated at 37 °C under agitation up to mid-log (OD600 ~ 0.5). A total of 200 μL of LB containing *E. coli* ER2738 (ECR) was added to each dilution of the phage for 5 min, and subsequently, each dilution was added to TOP agarose (LB 20 g/L, MgCl 2 6H_2_O 1 g/L, agarose 7 g/L) and labelled on LB agar plates.

### Inoculation in animals and collection of biological samples

The birds were inoculated at 2, 8, 15 days of age, receiving the treatment intraoesophagally. They were divided into five groups (28 birds/group), receiving the following treatments: Group G1 (G1) received 10 log PFU/bird of bacteriophage M13 in PEG and PBS; Group G2 (G2) was inoculated with 5 to 8 log CFU/bird of ECR infected with bacteriophage M13 diluted in PBS; Group G3 (G3) was inoculated with 6 to 8 log CFU/bird of ECR diluted in PBS; Group G4 (G4) received PEG (bacteriophage control); Group 5 (G5) was inoculated with PBS (negative control). The exact amounts of each inoculum of ECR are listed in Additional file 1: Table S3.

At 7, 14, 21 and 28 days of age, three birds of each group were euthanised, and we evaluated intestine integrity visually and collected faeces directly from the rectum to count the phages. We performed histomorphometry at 35 and 42 days of age, after macroscopic analyses. At 42 days of age, the blood of three birds was collected directly from the brachial vein (2 mL of blood), the gut was macroscopically analysed. The remaining birds were used only for zootechnical analysis.

### Phage count in faeces

In the Nanobiotechnology Laboratory at the Federal University of Uberlândia, the faeces were weighed and diluted in 5000 μL PBS (dilution 1:10), followed by homogenisation. Diluted faeces were filtered, and the remaining liquid was centrifuged at 15,000 × *g* for 10 min. The supernatant was spiked with 1/6 of PEG and stored in a cold chamber overnight (± 4 °C). Subsequently, the sample was centrifuged at 15,000 × *g* for 10 min, the pellet was resuspended, and 30 μL was placed in 9970 μL of LB; six serial dilutions were performed in LB. For labelling, the ECR was previously prepared in LB incubated at 37 °C under agitation up to mid-log (OD600 ~ 0.5). A total of 200 μL of LB containing ECR was added to each dilution of the phage for 5 min. Each dilution was added to TOP agarose and labelled in LB agar added to IPTG (isopropyl β-d-1-thiogalactopyranoside—Ludwig Biotec) (0.5 mM) in *mid-log* (OD600 ~ 0.5) and X-gal (5-bromo-4-chloro-3-indolyl β-d-galactopyranoside—Ludwig Biotec) (40 μg/mL), followed by incubation at 37 °C for 24 h. The blue colonies (where 15–50 colonies grew) on the plates were counted.

### DNA extraction

The blue colonies isolated from the faeces were amplificated in ECR and centrifugated at 15,000 × *g* RPM for 10 min. The supernatant was added to 1/6 of PEG overnight, and subsequently, the sample was centrifugated at 15,000 × *g* for 10 min; the obtained supernatant was resuspended in iodide buffer (10 mM Tris–HCl pH 8.0, 1 mM EDTA and 4 M NaI). The tubes were agitated for 40 s at ambient temperature, and 250 μL of ethanol was added. After 10 min, the phages were centrifuged at 3700 × *g* for 10 min at 20 °C, and the supernatant was removed. The DNA was washed with ethanol and centrifugated as described above. The precipitated DNA was diluted in nuclease-free water.

### PCR for M13 confirmation

Polymerase chain reaction (PCR) was performed to confirm that the blue colonies isolated from faeces were M13. The reactions were conducted for a final volume of 10 μL, containing 1 μL of the DNA sample, 10 pmol/μL of each primer (forward: 5ʹ-GTAAAACGACGGCCAG-3ʹ, reverse: 5ʹ-CAGGAAACAGCTATGAC-3ʹ) (Invitrogen), 0.25 μl of 20 mM of the mix of dNTPs (Invitrogen), 0.25 μL of Taq (5U/μL) (Invitrogen). The samples were submitted to the following amplification cycles: initial denaturation at 95 °C for 5 min, 35 cycles at 94 °C for 40 s, 56 °C for 40 s, 72 °C for 50 s, and a final extension at 72 °C for 10 min. All PCR reactions were performed on a thermal cycler (Eppendorf), and the amplified products were separated via 1.5% agarose gel electrophoresis. The gel was stained with Syber Safe (Invitrogen) and visualised in UV translucent vessels.

### Sequencing of M13 phage

To increase the certainty that the phages isolated were M13, we sequenced four blue colonies, using 200 ug of DNA, 5 pmol of primer-96 gIII (5ʹ-OH CCC TCA TAG TTA GCG TAA CG-3ʹ—Biolabs) and Premix (BigDye® Terminator v3.1 Cycle Sequencing Kit – Applied). The 35 cycles were performed in a plate thermocycler (MasterCycler-Eppendorf) under the following conditions: denaturation (95 °C, 20 s), annealing (50 °C, 40 s) and extension (60 °C, 60 s). The amplified DNA was resuspended in 10 μL of dilution buffer, and the sequences were read on a ABI3730xl Genetic Analyzer (Applied Biosystems). The DNA sequences were analysed using the  the Chromas Lite program (https://chromas-lite.software.informer.com/2.1/). Immediately after this pre-analysis, the vector sequences were removed, and only those inserted with perfect residues were translated using the Expasy translate program (https://web.expasy.org/translate/).

### Performance and macroscopic and gut macroscopic change analysis

The birds were monitored two times a day, and the typical behaviour (activity, scratching, opening the wings when bathing in the shavings) as well as the faeces characteristics were evaluated. The birds were weighed at 1, 7, 35 and 42 days of age. Uniformity was calculated, and feed intake and conversion were calculated based on the weighed rations. At 7, 14, 21, 28 and 42 days of age, three birds of each group were euthanised and necropsy was performed, observing all organs in the cloacal pouch, thymus, spleen, liver, oesophagus, crop, gizzard, intestine and cecal tonsils.

### Intestine histomorphometric analyses

At 35 and 42 days of age, the ileum and cecum of the G2 and G5 were collected for histomorphometric analysis. The fragments were fixed in 10% buffered formalin and processed to prepare histological slides stained with haematoxylin and eosin (HE) (Tolosa et al. 2003). Ileum villi height and ileum and cecum crypt depth were measured using the ImageJ morphometry program.

### ELISA

To assess whether the phages were able to produce a systemic immune response, ELISA was performed at 42-day-old birds as described (Santos et al. 2018), with modifications. The microtitration plates were sensibilised with 1 μg/well of phage M13 diluted in 50 mM bicarbonate buffer (pH 8.6) for 16 h at 4 °C. After three washes with PBS-T (PBS + Tween 20 at 0.05%), the plates were blocked with PBS-T with 5% skim powdered milk for 1 h at 37 °C and subsequently washed three times with PBS-T, followed by incubation with different bird serum at different dilutions (1:50 and 1:100) at 37 °C for 1 h. The controls were performed without phages or serum. After five washes with PBS-T, the anti-IgY was added, followed by incubation at 37 °C for 1 h. The wells were washed five times, and the ligation antigen/antibody was detected by the addition of ortho-phenylenediamine (OPD) at 1 mg/mL with 3% H_2_O_2_ (Sigma Chemical Co.). The reaction was stopped by the addition of 4 N sulfuric acid. Reactivity was determined in a plate reader (Titertek Multiskan Plus, Flow Laboratories, USA) at a wavelength of 492 nm. The results represented the absorbance in wells sensitised with phage subtracted from that of the control wells (not sensitised with phages).

### Statistical analysis

We performed analysis of variance (ANOVA) followed by Tukey's test to analyse the ELISA and phage counts or test t to histomorphometric, with a 95% confidence interval, using the GraphPad Prism 9.2. We used the GLM (General Linear Models) procedure of the SAS statistical package (2008) to weigh gain analysis.

## Results

### It is necessary the ECR to M13 viability in poultry

#### Quantification of plaque-forming units (PFUs)

We inoculated phage M13 and ECR infected with phage M3 into 2-, 8- and 15-day-old birds. However, we only recovered M13 in the faeces of the group treated with ECR infected by phage M13 (Group 2) (Table 1).

**Table 1:** Mean values of M13 phages per gram of bird faeces (log PFU/g) in different groups and at different ages

Age/Groups	G1	G2	G3	G4	G5
7	0.00 (± 0.00)^a^	4.84 (± 0.82)^b^	0.00 (± 0.00)^a^	0.00 (± 0.00)^a^	0.00 (± 0.00)^a^
14	0.00 (± 0.00)^a^	5.27 (± 0.25)^b^	0.00 (± 0.00)^a^	0.00 (± 0.00)^a^	0.00 (± 0.00)^a^
21	0.00 (± 0.00)^a^	5.77 (± 0.57)^b^	0.00 (± 0.00)^a^	0.00 (± 0.00)^a^	0.00 (± 0.00)^a^
28	0.00 (± 0.00)^a^	4.6 (± 0.60)^b^	0.00 (± 0.00)^a^	0.00 (± 0.00)^a^	0.00 (± 0.00)^a^

Values represent mean and standard deviation. G1: group inoculated with phage M13. G2: Group inoculated with *E. coli* ER2738 infected by the phage M13; G3: Group infected with *E. coli* ER2738; G4: Group inoculated with PEG; G5: Group inoculated with PBS. The birds of G1 were inoculated at all ages (10 PFUs/bird). The birds of G3 were inoculated at 2 days (5.04 log CFU/animal), 8 days (6.64 log CFU/animal) and 15 days of age (6.69 log CFU/animal). Different letters represent statistical difference (P > 0.05) by grouped ANOVA

#### PCR of t phage M13 suspect colonies

As we had no information about the microbiota of the poultry studied, we performed a PCR to confirm that the colonies found were the M13 phage. Based on the results, the phages found amplified with the phage primers used.

#### Sequencing the M13 phage

Sequencing was performed to confirm that the colonies were the M13 phages from the PD library. The sequences of all four blue colonies were identical, and the analysis confirmed the M13 phage from the PD library. In all results, the sequence 'CTATTCTCAC', located in the P3 insert region of the phage M13, was present.

### The presence of phage M13 in the gut does not change zootechnical parameters or cause damage to poultry

The birds were monitored twice daily. We observed no changes in their behaviour and health. Via necropsy of euthanised animals, we found no organ changes. During the experiment, the mortality rate was zero; there were no changes in weight and average daily feed intake (Table 2).

**Table 2:** Mean daily weight gain of birds (MDWG), mean daily feed consumption (MDFC) and feed conversion (FA) of bords of the line Hy-line W-36® at 1–7 days, 8–35 days and 36–42 days of age

Tratament^1^	1–7 days old (g/day)			8–35 days old (g/day)			36–42 days old (g/day)		
	MDWG	MDFC^2^	FA^2^	MDWG	MDFC^2^	FA^2^	MDWG	MDFC^2^	FA^2^
G 1	14.29	19.80	1.39	31.76	46.20	1.45	57.37	139.46	2.43
G 2	15.59	19.83	1.27	30.64	46.16	1.51	59.78	132.89	2.22
G 3	15.61	19.58	1.25	30.00	50.74	1.69	47.87	132.43	2.77
G 4	14.97	20.42	1.36	27.88	41.10	1.47	68.09	117.17	1.72
G 5	15.86	18.89	1.19	31.16	45.04	1.45	61.99	135.17	2.18
Mean	15.26	19.70	1.29	30.29	45.85	1.51	59.02	131.42	2.26
CV (%)^3^	9.37	–	–	9.69	–	–	7.66	–	–
*P-valor*^4^	0.21	–	–	0.64	–	–	0.51	–	–

G1: group inoculated with phage M13. G2: Group inoculated with *E. coli* ER2738 infected by the phage M13; G3: Group infected by *E. coli* ER2738; G4: Group inoculated with PEG; G5: Group inoculated with PBS. The birds of G1 were inoculated at all ages (10 PFUs/bird). The birds of G3 were inoculated at 2 days (5.04 log CFU/animal), 8 days (6.64 log CFU/animal) and 15 days of age (6.69 log CFU/animal). ^1^Means corresponding to the experimental treatments; ^2^MDFC and FA data are descriptive only; ^3^CV (%) corresponds to the coefficient of variation; ^4^Statistical probability; no differences were found for the MDWG variable (P > 0.05) by the Tukey test

### The presence of M13-infected ECR did not change the intestinal histomorphometry of ileum and cecum

**Fig. 1:**
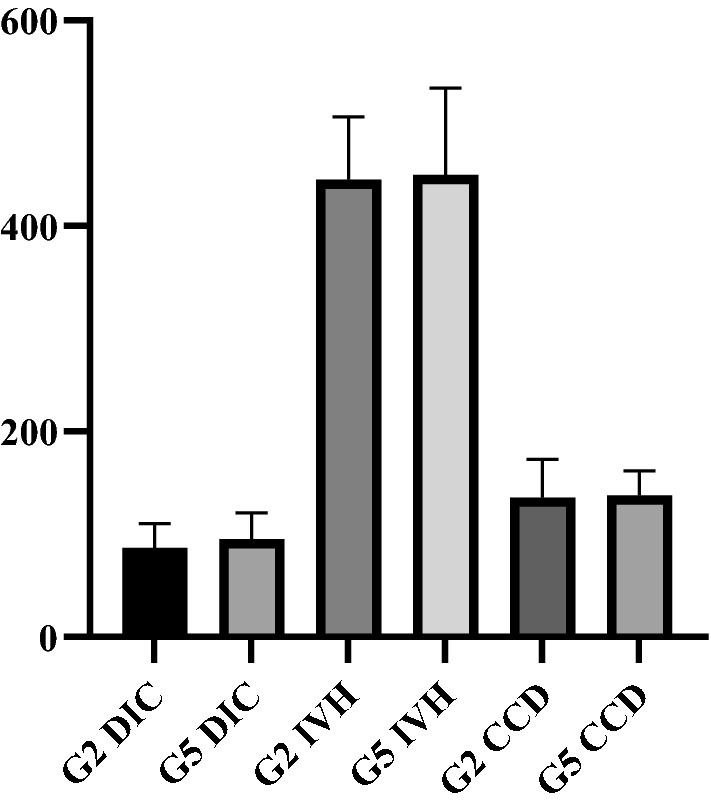
Ileum villi height and ileum and cecum crypt depth (um) in 42-day-old birds inoculated with M13-infected ECR. G2: Group inoculated with E. coli ER2738 infected by phage M13; G5: Group inoculated with PBS. DIC: depth of the ileum crypts, IVH: ileum villi height, CCD: cecum crypt depth. T tests were performed between G2 and G5 to DIC or IVH or CCD (p ≤ 0.05). There was no difference in the DIC, IVH or CCD between G2 and G5

We performed histomorphometric analyses of the ileum and cecum. No microscopic changes related to inflammation, degeneration or necrosis were observed. Furthermore, ileum villi heigh or the depth of the cecum and ileum crypts did not change (Fig. 1).

### Birds did not develop a humoral immune response against M13 phage

**Fig. 2:**
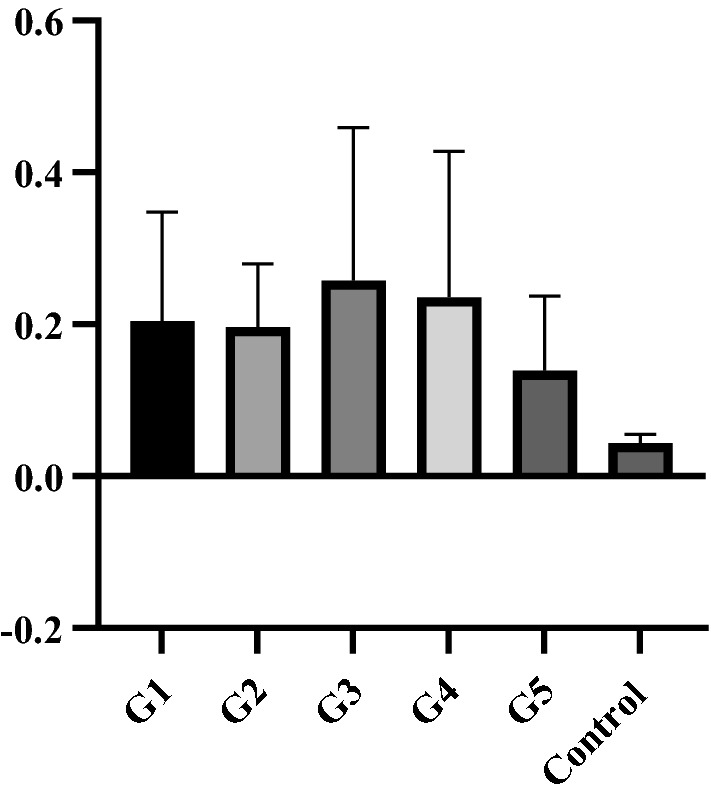
Levels of antibody (measured by absorbance) anti-M13 in birds of the different treatment groups. G1: Group inoculated with phage M13; G2: Group inoculated with *E. coli* ER2738 infected with phage M13; G3: Group infected by *E. coli* ER2738; G4: Group inoculated with PEG; G5: Group inoculated with PBS. Control: plate sensitised with phage without serum. No differences were found (P > 0.05) by Tukey’s test

To assess whether the phages could induce a systemic immune response, ELISA was carried out in 42-day-old birds. There were no differences in the ELISA results among the groups (Fig. 2).

## Discussion

Alternatives to the use of antibiotics in animal production are urgently needed. In this sense, several molecules are tested and used in poultry production. The PD technology allows the selection of peptides with high affinity and specificity to the antigen of interest. It is also suitable for applications in different research areas, being of paramount importance for innovation in diagnosing and treating various diseases (Henry et al. 2015).

Based on our results, phage M13 is viable inside the poultry gut and can replicate, but only when inoculated with ECR. Our hypothesis about the replication is based on the quantification of M13 per gram of faeces. Five or 6 days after inoculation, the amounts of the phage M13 per gram of faeces were 0.91, 1.25, 1.14 log PFU above the inoculated amount of ECR infected by M13 (Additional file 1: Table S3). Besides, 13 days after the last inoculation (at 28 days of age), the birds had 1.2 log PFU/g of faeces above the inoculated amount of ECR infected by M13 (Additional file 1: Table S3). Under laboratory conditions, M13 takes 5 min to invade the bacterium (Barbas et al. 2001). As we inoculated the infected bacteria, the phages then replicated within the bacteria and reached the intestinal tract, probably performing other cycles. We collected samples for analysis at 42 days, but unfortunately, an equipment issue did not allow us to obtain the results.

Interestingly, we found no M13 in birds inoculated without bacterium. Phage M13 needs an *E. coli* with sexual pili to replicate (Chung et al. 2011). Although *E. coli* commonly occurs in the microbiota on the intestine, not all *E. coli* have sexual pili. The specificity of the phage M13 to ECR is a critical point since this feature does not allow this phage to spread the resistance gene from the sexual pili.

Bacteriophage M13 can remain viable in the gut poultry; we recovered free phages from the faeces when inoculating ECR infected with M13. However, we do not know whether the M13 phage was found in the faeces (when inoculated alone) because it did not survive at the conditions of the gastric tract or because there was no *E. coli* with sexual pili in the intestine of the poultry. However, in practical terms, if we are going to use phage M13 displaying peptides binding to some antibody or antigen in the intestine, it is necessary to use *E. coli* expressing sexual pilli*.* Or else, technologies should be applied to ensure mucoadhesion and/or delivery and persistence of the M13 phage in the gastrointestinal tract over a long period.

When we started the experiment, our main concern was to isolate lactose-fermenting bacteria from the microbiota or even other phages that could infect *E. coli*. To ensure this, we performed several rounds of phage purification, PCR of 100% of the countable colonies and sequenced four of them. The PCR results showed that we had isolated phage M13. We used a primer that loops in the PIII-fused amino acid insert region in the sequencing, which made us more confident that we could isolate the inoculated phages from the faeces.

When analysing a probiotic microorganism or even a phage in animals, it is essential to assess virulence. Therefore, we evaluated several bird parameters such as behaviour, weight gain, morbidity, mortality, as well as macroscopic and microscopic lesions. Our analysis showed that the phage is safe for the birds because neither zootechnical, macroscopic, microscopic and histomorphometric changes were observed (Table 2, Fig. 1). One of the important issues with phage therapy is the development of immunity against the phage (Krut and Bekeredjian-Ding 2018); therefore, we performed the ELISA at 42 days of age. Based on the results, there was no increase in antibodies against M13 in the treated group, indicating that in birds with a short cycle, phage M13 can be used without the bird's organism producing antibodies that inactivate this virus.

Inoculation of ECR infected with bacteriophage M13 allows this phage to survive in the intestines of chickens without harming them. This could be a new alternative to the use of PD for the control of pathogens in poultry.

## Supplementary Information

**Additional file 1: Table S1.** Nutritional requirements of birds and vitamin and mineral supplementation. **Table S2.** Ingredient compositions of initial (1–7 days old), fattening (7–32 days old) and finishing (33–42 days) diets for birds. **Table S3.** Quantification of ECR and phage M13 inoculated in birds and phage M13 isolated from bird faeces.

## Data Availability

Data are contained within the article or the Additional Material. Complete data will be made available upon request.
