# Correction: Alternative use of phage display: phage M13 can remain viable in the intestines of poultry without causing damage

**DOI:** 10.1186/s13568-022-01427-5

**Published:** 2022-07-09

**Authors:** Fabiana de Almeida Araújo Santos, Edson Campos Valadares Junior, Luiz Ricardo Goulart, Pedro Lucas Figueiredo Nunes, Eliane Pereira Mendonca, Lucio Vilela Carneiro Girao, Aline Santana da Hora, Thatiana Bragine Ferreira, Luciana Machado Bastos, Alessandra Aparecida Medeiros‑Ronchi, Belchiolina Beatriz Fonseca

**Affiliations:** 1 Faculdade de Medicina Veterinaria, Universidade Federal de Uberlandia, Uberlandia, Brazil; 2 Instituto de Biotecnologia da Universidade Federal de Uberlandia, Uberlandia, Brazil; 3 Pos-Graduacao em Medicina Tropical e Infectologia da Universidade Federal do Triangulo Mineiro, Uberaba, Brazil

## Correction to: AMB Express (2022) 12:64 https://doi.org/10.1186/s13568-022-01407-9

Following publication of the original article (Santos et al. 2022), the authors reported an error in the first author’s name “Fabiana de Almeida Araújo Santos”. The given name should be “Fabiana de Almeida Araújo” and last name is “Santos”. This has been corrected with this erratum.

The original article (Santos et al. 2022) has been updated.
