# Supplementary material for: Alternative use of phage display: phage M13 can remain viable in the intestines of poultry without causing damage
Source: AMB Express. 2022 Jun 2;12:64. doi: 10.1186/s13568-022-01407-9 (PMC9160166; doi:10.1186/s13568-022-01407-9)
Supplement: Supplementary file 1 — Additional file 1: Table S1. Nutritional requirements of birds and vitamin and mineral supplementation. Table S2. Ingredient compositions of initial (1–7 days old), fattening (7–32 days old) and finishing (33–42 days) diets for birds. Table S3. Quantification of ECR and phage M13 inoculated in birds and phage M13 isolated from bird faeces. [file 13568_2022_1407_MOESM1_ESM.docx]

**Alternative use of *phage display*: Phage M13 can remain viable in the intestines of poultry without causing damage**

Fabiana de Almeida Araújo Santos^1#^. Edson Campos Valadares Junior^2#^. Luiz Ricardo Goulart^2£^. Pedro Lucas Figueiredo Nunes^2^. Eliane Pereira Mendonça^2^. Lúcio Vilela Carneiro Girão^2^. Aline Santana da Hora^1^. Thatiana Bragine Ferreira^3^. Luciana Machado Bastos^1^. Alessandra Aparecida Medeiros-Ronchi.^2^ Belchiolina Beatriz Fonseca^1.2*^

1. Faculdade de Medicina Veterinária Universidade Federal de Uberlândia. Uberlândia, Brasil.
2. Instituto de Biotecnologia da Universidade Federal de Uberlândia. Uberlândia, Brasil.
3. Pós-graduação em Medicina Tropical e Infectologia Universidade Federal do Triângulo Mineiro. Brasil

# These authors share the first authorship as they contribute equally to the article

^£.^ *In memoriam*

*Corresponding author: biafonseca@ufu.br

**Supplementary Material**

Table S1. Nutritional requirements of birds and vitamin and mineral supplementation.

| **NUTRIENT** | Initial | | Fattening | | Finishing | |
| --- | --- | --- | --- | --- | --- | --- |
|  | 1–7 (days old) | | 7–32 (days old) | | 33–42 (days old) | |
| Metabolisable energy (Kcal/kg)^1^ | 3.000 | | 3.100 | | 3.100 | |
| Crude protein (%)^1^ | 20.79 | | 19.41 | | 19.41 | |
| Calcium (%)^2^ | 0.88 | | 0.82 | | 0.82 | |
| Phosphorus (%)^2^ | 0.44 | | 0.41 | | 0.41 | |
| Sodium (%)^2^ | 0.21 | | 0.21 | | 0.21 | |
| Potassium (%)^2^ | 0.59 | | 0.59 | | 0.59 | |
| Chlorine (%)^2^ | 0.19 | | 0.18 | | 0.18 | |
| Linoleic acid (%)^2^ | 0.63 | | 0.57 | | 1.11 | |
| Amino acid^2^ | Dig. | Total | Dig. | Total | Dig. | Total |
| Lysine (%)^2^ | 0.98 | 1.07 | 0.88 | 0.96 | 0.76 | 0.83 |
| Methionine (%)^2^ | 0.44 | 0.47 | 0.40 | 0.44 | 0.36 | 0.38 |
| Methionine + cystine (%)^2^ | 0.74 | 0.83 | 0.67 | 0.75 | 0.59 | 0.67 |
| Tryptophan (%)^2^ | 0.18 | 0.21 | 0.17 | 0.20 | 0.15 | 0.18 |
| Threonine (%)^2^ | 0.66 | 0.77 | 0.60 | 0.70 | 0.52 | 0.62 |
| Arginine (%)^2^ | 1.05 | 1.13 | 0.94 | 1.01 | 0.81 | 0.87 |
| Valine (%)^2^ | 0.73 | 0.80 | 0.69 | 0.76 | 0.61 | 0.67 |
| Isoleucine (%)^2^ | 0.71 | 0.76 | 0.65 | 0.70 | 0.57 | 0.61 |
| Vitamin A (UI/kg)^2^ | 12,000 | | 10,000 | | 9,000 | |
| Vitamin D3 (UI/kg)^2^ | 5,000 | | 4,500 | | 4,000 | |
| Vitamin E (UI/kg)^2^ | 80 | | 65 | | 55 | |
| Vitamin K (mg/kg)^2^ | 3.2 | | 3.0 | | 2.2 | |
| Vitamin C (mg/kg)^2^ | 50 | | 50 | | 25 | |
| Folic acid (mg/kg)^2^ | 2.2 | | 1.9 | | 1.6 | |
| Biotin (mg/kg)^2^ | 22 | | 18 | | 15 | |
| Choline (mg/kg)^2^ | 1,700 | | 1,600 | | 1,550 | |
| Manganese (mg/kg)^2^ | 120 | | 120 | | 120 | |
| Zinc (mg/kg)^2^ | 110 | | 110 | | 120 | |
| Iron (mg/kg)^2^ | 20 | | 20 | | 20 | |
| Copper (mg/kg)^2^ | 16 | | 16 | | 16 | |
| Selenium (mg/kg)^2^ | 0.30 | | 0.30 | | 0.30 | |
| Iodine (mg/kg)^2^ | 1.25 | | 1.25 | | 1.25 | |
| ^1^ Levels calculated according to the recommended formulation | | | | | |  |
| ^2^ Levels calculated according to the genetic lineage recommendation (Hi-Line 2016) | | | | | |  |

| Table S2. Ingredient compositions of initial (1–7 days old), fattening (7–32 days old) and finishing (33–42 days) diets for birds. | | | |  |
| --- | --- | --- | --- | --- |
| **INGREDIENTS^1^** | Initial (g/kg) | Fattening (g/kg) | Finishing (g/kg) | |
|  | 1–7 (days old) | 7–32 (days old) | 33–42 (days old) | |
| Corn meal 7.88% | 593.2 | 614.3 | 614.3 | |
| Soybean meal 45% | 344.3 | 308.4 | 308.6 | |
| Soybean oil | 23.00 | 32.6 | 32.7 | |
| Dicalcium phosphate | 18.00 | 16.7 | 16.7 | |
| Limestone | 9.00 | 8.4 | 8.4 | |
| DL-Methionine 99% | 1.55 | 1.55 | 1.34 | |
| L-Lysine HCl 78.4% | 1.85 | 2.03 | 2.03 | |
| L-Threonine 98.5% | 0.5 | 0.5 | 0.5 | |
| Choline chloride 70% | 0.5 | 0.5 | 0.5 | |
| Sodium chloride | 4.95 | 4.7 | 4.7 | |
| Anticoccidian^2^ | -- | -- | -- | |
| Antibiotic^2^ | -- | -- | -- | |
| Vitamin/mineral supplement | 3.0 | 3.0 | 3.0 | |
| Total (g) | 1,000 | 1,000 | 1,000 | |

^1^ Levels calculated according to the genetic lineage recommendation (Hi-Line 2016)

^2^ Not included for influencing the results

Table S3. Quantification of ECR and phage M13 inoculated in birds and phage M13 isolated from bird faeces.

| **Initial inoculum of infected bacterium** | | **Quantification of phage M13 in faeces** | | **Difference between last inoculum and phage M13 in faeces** |
| --- | --- | --- | --- | --- |
| **Age (days)** | **log CFU/bird** | **Age** | **log PFU/g of faeces** | **Log CFU** |
| 2 | 5.04 | 7 | 3.93 | 1.11 |
| 8 | 6.64 | 14 | 4.02 | 2.62 |
| 15 | 6.69 | 21 | 4.36 | 2.33 |
|  |  | 42 | 3.40 | 3.29 |

ECR: *E. coli* ER2738V
